# In vitro activity of tedizolid against 43 species of *Nocardia* species

**DOI:** 10.1038/s41598-024-55916-7

**Published:** 2024-03-04

**Authors:** Masahiro Toyokawa, Noboru Ohana, Daiki Tanno, Minako Imai, Yukiko Takano, Kazutaka Ohashi, Tomonari Yamashita, Kyoichi Saito, Hiroki Takahashi, Hiroki Shimura

**Affiliations:** 1 Department of Clinical Laboratory Sciences, School of Health Sciences, Fukushima Medical University, Fukushima, 10-6, Sakaemachi, Fukushima City, Fukushima 960-8516 Japan; 2 Department of Laboratory Medicine, Fukushima Medical University, 1 Hikariga-Oka, Fukushima City, Fukushima 960-1295 Japan; 3 Department of Clinical Laboratory Medicine, Fukushima Medical University Hospital, 1 Hikariga-Oka, Fukushima City, Fukushima 960-1295 Japan; 4 Clinical Testing Department, MicroSKY Lab, Inc., Center Building Kanamachi 2F, 6-6-5 Higashikanamachi, Katsushika-ku, Tokyo, 125-0041 Japan; 5 Medical Mycology Research Center, Chiba University, 1-8-1 Inohana, Chuo-ku, Chiba, 260-8673 Japan; 6 Molecular Chirality Research Center, Chiba University, 1-33 Yayoi-cho, Inage-ku, Chiba, 263-8522 Japan; 7 Plant Molecular Science Center, Chiba University, 1-8-1 Inohana, Chuo-ku, Chiba, 260-8675 Japan

**Keywords:** *Nocardia* species, In vitro activity, Tedizolid, Linezolid, Resistance mechanisms, Antimicrobials, Clinical microbiology

## Abstract

The purpose of the present study was to evaluate the in vitro activity of tedizolid against several clinically significant species of *Nocardia* by comparing with that of linezolid. A total of 286 isolates of *Nocardia* species, including 236 clinical isolates recovered from patients in Japan and 50 strains (43 species) purchased from NITE Biological Resource Center, were studied. Antimicrobial susceptibility testing was performed using the broth microdilution method. For the 286 *Nocardia* isolates, the minimal inhibitory concentration (MIC)_50_ and MIC_90_ values of tedizolid were 0.25 and 0.5 μg/ml, and those of linezolid were 2 and 2 μg/ml, respectively. The distribution of the linezolid/tedizolid ratios (MICs of linezolid/MICs of tedizolid) showed that tedizolid had four- to eight-fold higher activity than linezolid in 96.1% (275/286) of *Nocardia* isolates. Both the tedizolid and linezolid MIC_90_ values for *Nocardia brasiliensis* were two-fold higher than those for the other *Nocardia* species. Both tedizolid and linezolid had low MIC values, 0.25–1 μg/ml and 0.5–4 μg/ml, respectively, even against nine isolates (five species) that were resistant to trimethoprim/sulfamethoxazole. One *Nocardia sputorum* isolate showed reduced susceptibility to tedizolid (4 μg/ml). Bioinformatics analysis suggests different resistance mechanisms than the oxazolidinone resistance seen in enterococci and staphylococci.

## Introduction

*Nocardia* species are soilborne aerobic actinomycetes that can cause opportunistic infections in humans. Nocardiosis is typically seen in immunocompromised patients with a history of solid organ transplantation, hematopoietic stem cell transplantation, malignancy, or chronic pulmonary diseases; however, it may also occur in immunocompetent individuals^1^. The mortality rate of disseminated nocardiosis in hematopoietic cell transplantation recipients is high^2^. A rapid and reliable method for the detection of the causative microorganisms in clinical specimens is essential in order to reduce mortality and expedite appropriate antimicrobial therapy^3^. The antimicrobial treatment of nocardiosis requires that antibiotics are administered for several months^4^. Trimethoprim-sulfamethoxazole has long been considered the therapeutic agent of choice, either alone or in combination with amikacin or beta-lactams^4^. However, some clinically relevant species of *Nocardia* are multidrug resistant, including trimethoprim/sulfamethoxazole^5–7^, and there is a paucity of other antimicrobials with sufficient efficacy against these organisms. Thus, new antimicrobials with therapeutic potential are desperately needed.

Linezolid has been used effectively in the therapy of patients with nocardiosis; however, side effects, such as peripheral neuropathy and myelosuppression, have been reported during long-term use in some patients^8^. On the other hand, new oxazolidinone compounds such as tedizolid have shown excellent activity both in vitro and in vivo against *Nocardia brasiliensis*^9,10^. It has also been reported that tedizolid has a low rate of myelosuppression during long-term treatment^11^ and has activity against linezolid-resistant *Staphylococcus aureus* and enterococci^12–14^.

The purpose of the present study was to evaluate the in vitro activity of tedizolid against several clinically significant species of *Nocardia* compared with those of linezolid.

## Materials and methods

### Bacterial isolates and identification

A total of 286 strains of *Nocardia* species, including 236 clinical isolates (15 species/complexes) recovered from patients in 27 microbiology laboratories in Japan and 50 strains (43 species) purchased from the NITE Biological Resource Center (NBRC) (National Institute of Technology and Evaluation, Tokyo, Japan), were studied (Table 1). Identification of *Nocardia* species was based on Gram-stain, colonial morphology, and molecular technique. Species identification of all clinical isolates was performed by Matrix-Assisted Laser Desorption/Ionization Time-of-Flight Mass Spectrometry (microflex LT, Bruker Daltonics)^15^ and by full-length 16S rRNA gene sequencing^16^. *Nocardia* spp. that were difficult to identify via 16S rRNA gene sequencing were classified as a complex^17,18^. Two strains of new species of *N. sputorum* (IFM 12275, IFM 12276^T^), which were previously reported as a novel species by our group^19^, were included in the *N. abscessus* complex. These strains were closely related to *N. beijingensis* NBRC16342^T^ (99.6%) by phylogenetic analysis based on the 16S rRNA sequence^19^. The GenBank/EMBL/DDBJ accession numbers for the 16S rRNA gene sequence and the complete genome sequence of strains IFM 12276^T^ and IFM 12275 are LC741024 and AP026978, and LC741023 and AP026976 (plasmid: AP026977), respectively.

**Table 1:** Species distribution of the 286 *Nocardia* strains subjected in this study.

Species	No. of isolates		
	Clinical isolates (n = 236)	NBRC^a^ strains (n = 50)	NBRC No
*N. farcinica* complex^b^	61		
*N. farcinica*		6	NBRC15532^T^, NBRC3384, NBRC3423, NBRC3424, NBRC3927, NBRC13510
*N. cyriacigeorgica*	45	1	NBRC100375^T^
*N. nova* complex^c^	37		
*N. nova*		2	NBRC103080, NBRC15556^T^
*N. elegans*		1	NBRC108235^T^
*N. aobensis*		1	NBRC100429^T^
*N. africana*		1	NBRC100379^T^
*N.kruczakiae*		1	NBRC101016^T^
*N. veterana*		1	NBRC100344^T^
*N. vermiculata*		1	NBRC100427^T^
*N. abscessus* complex^d^	35		
* N. abscessus*		1	NBRC100374^T^
* N. asiatica*		1	NBRC100129^T^
* N. beijingensis*		1	NBRC16342^T^
* N. arthritidis*		1	NBRC100137^T^
* N. sputorum*			NBRC115476, NBRC115477^T^
*N. brasiliensis*	17	1	NBRC14402^T^
*N. transvalensis* complex^e^	15		
* N. transvalensis*		1	NBRC15921^T^
*N. otitidiscaviarum*	12	1	NBRC14405^T^
*N. thailandica*	2	1	NBRC100428^T^
*N. takedensis*	1	2	NBRC100417^T^, NBRC100418
*N. asteroides*	1	1	NBRC15531^T^
*N. vinacea*	1	1	NBRC16497^T^
*N. mexicana*	1	1	NBRC108244^T^
*N. niigatensis*	1	1	NBRC100131^T^
*N. pseudobrasiliensis*	1	1	NBRC108224^T^
*N. yamanashiensis*	1	1	NBRC100130^T^
*N. araoensis*		1	NBRC100135^T^
*N. paucivorans*		1	NBRC100373^T^
*N. brevicatena*		1	NBRC12119^T^
*N. anaemiae*		1	NBRC100462^T^
*N. carnea*		1	NBRC14403^T^
*N. concava*		1	NBRC100430^T^
*N. exalbida*		1	NBRC100660^T^
*N. higoensis*		1	NBRC100133^T^
*N. ignorata*		1	NBRC108230^T^
*N. inohanensis*		1	NBRC100128^T^
*N. neocaledoniensis*		1	NBRC108232^T^
*N. ninae*		1	NBRC108245^T^
*N. pneumoniae*		1	NBRC100136^T^
*N. puris*		1	NBRC108233^T^
*N. shimofusensis*		1	NBRC100134^T^
*N. sienata*		1	NBRC100364^T^
*N. terpenica*		1	NBRC100888^T^
*N. testacea*		1	NBRC100365^T^
*N. uniformis*		1	NBRC13702^T^
*Nocardia* sp.	5		
Total	236	50	

^a^NITE Biological Resource Center (NBRC), ^b^*N. farcinica* and *N. kroppenstedtii* were included in the *N. farcinica* complex, ^c^*N. nova*, *N. elegans*, *N. aobensis*, and *N. veterana* were included in the *N. nova* complex, ^d^*N. abscessus*, *N. asiatica*, *N. beijingensis*, *N. sputorum*, and *N. arthritidis* were included in the *N. abscessus* complex, ^e^*N. wallacei*, *N. transvalensis*, and *N. blacklockiae* were included in the *N. transvalensis* complex.

### Antimicrobial susceptibility testing (AST)

Antimicrobial Susceptibility Testing (AST) of 286 *Nocardia* isolates was performed using the broth microdilution method with frozen panels (Eiken Chemical, Tokyo, Japan), according to the Clinical and Laboratory Standards Institute (CLSI) M24-A3 guidelines^20^. In brief, a heavy organism suspension was prepared in a small volume of sterile saline with 7–10 3-mm glass beads and was vortexed vigorously. Clumps were allowed to settle for 15 min, and the supernatant was adjusted to a 0.5 McFarland standard using a calibrated nephelometer. For frozen panel inoculation, the adjusted 0.5 McFarland suspension was diluted 30-fold with sterile saline, and 10 µl of the diluted solution was dispensed into each well of the panel. The panels were incubated at 35 °C for 48–72 h and, if needed, daily thereafter for up to 5 days. For trimethoprim/sulfamethoxazole, the MICs were determined as the wells corresponding to 80% inhibition of growth compared to the controls. The MICs were determined for tedizolid, linezolid, trimethoprim/sulfamethoxazole, amikacin, tobramycin, clarithromycin, amoxicillin-clavulanic acid, ciprofloxacin, moxifloxacin, minocycline, imipenem, and ceftriaxone on the same panel, and were interpreted as recommended by CLSI. For this analysis, *Staphylococcus aureus* ATCC 29213 and *Nocardia nova* ATCC BAA-2227 were used as the quality control strains.

For determination of trimethoprim/sulfamethoxazole resistance, disk diffusion testing with a 250-μg sulfisoxazole disk (Hardy Diagnostics, CA, USA) was performed according to the CLSI M24-A3 guidelines^20^. For this analysis, *N. nova* ATCC BAA-2227 and *Escherichia coli* ATCC 25922 were used as the quality control strains.

### Minimum bactericidal concentration testing

Minimum bactericidal concentration (MBC) testing for tedizolid and linezolid against 23 clinical isolates (eight of *Nocardia farcinica* complex, four of *Nocardia cyriacigeorgica*, three of *N. brasiliensis*, three of *N. nova* complex, two of *Nocardia abscessus* complex, two of *Nocardia transvalensis* complex, and one of *Nocardis* sp.) was performed according to the Clinical Microbiology Procedures Handbook^21^. The isolates were selected from species that are frequently isolated from clinical materials. The MBC was defined as the lowest antibiotic concentration able to kill at least 99.9% (reduction of 3 log10) of the initial inoculum.

### Analysis of resistance mechanisms in tedizolid-resistant isolate

Mechanisms of tedizolid resistance in IFM 12275, which showed reduced susceptibility to tedizolid (4 μg/ml), were investigated by comparison with those reported in enterococci^22–26^ and staphylococci^12–14^. The same analysis was also performed for IFM 12276^T^, which was susceptible to tedizolid (0.25 μg/ml) as a control strain. Specifically, acquired linezolid resistance genes (*cfr*, *cfr*(B), *cfr*(D), *optrA*, *poxtA*^25^) were analysed using ARG-ANNOT (V6, https://ifr48.timone.univ-mrs.fr/blast/arg-annot_v6.html) and CARD (https://card.mcmaster.ca/) software. Primer-BLAST analysis (https://www.ncbi.nlm.nih.gov/tools/primer-blast/index.cgi?LINK_LOC=BlastHome) using the primer sequences shown in Table S1 was also conducted.

Analysis of nucleotide mutations within the 23SrRNA gene mutations (G2576T, G2505A, U2500A, G2447U, G2534U, G2603U: *E. coli* J01695 numbering^22^) in IFM 12275 and IFM 12276^T^ were performed as follows. Briefly, the 23SrRNA gene from *N. farcinica* strain IFM10152 (https://www.ncbi.nlm.nih.gov/nuccore/NR_076254.1) was used as the reference sequence, and each sequence of the 23SrRNA gene in IFM 12275 and IFM 12276^T^ was mapped to the reference gene by the Nucleotide BLAST (https://blast.ncbi.nlm.nih.gov/Blast.cgi?PROGRAM=blastn&PAGE_TYPE=BlastSearch&LINK_LOC=blasthome). Position numbering, position converted by *E. coli* J01695 number, was used according to recommendation^27^.

Analysis of mutations in the 50S ribosomal proteins, L3 (*rplC*), L4 (*rplD*), and L22 (*rplV*)^22,24^, were performed for comparing homology between their sequences of IFM 12275 and IFM 12276^T^ strains annotated using the annotation pipeline Prokka v.1.14.6^28^. The orthologous gene sets among IFM 12275 and IFM 12276^T^ strains were detected by Panaroo v.1.2.10^29^.

### Ethical statement

The present study was conducted in accordance with the ethical guidelines of the Ministry of Health, Labour and Welfare, Japan. No ethical committee approvals or informed consent were needed for this study.

### Conference presentation

A part of this study was presented at the 32nd European Congress of Clinical Microbiology and Infectious Diseases, 23 to 26 April 2022, Lisbon, Portugal [abstr. #00218].)

## Results

### Antimicrobial Susceptibility Testing

**Figure 1:**
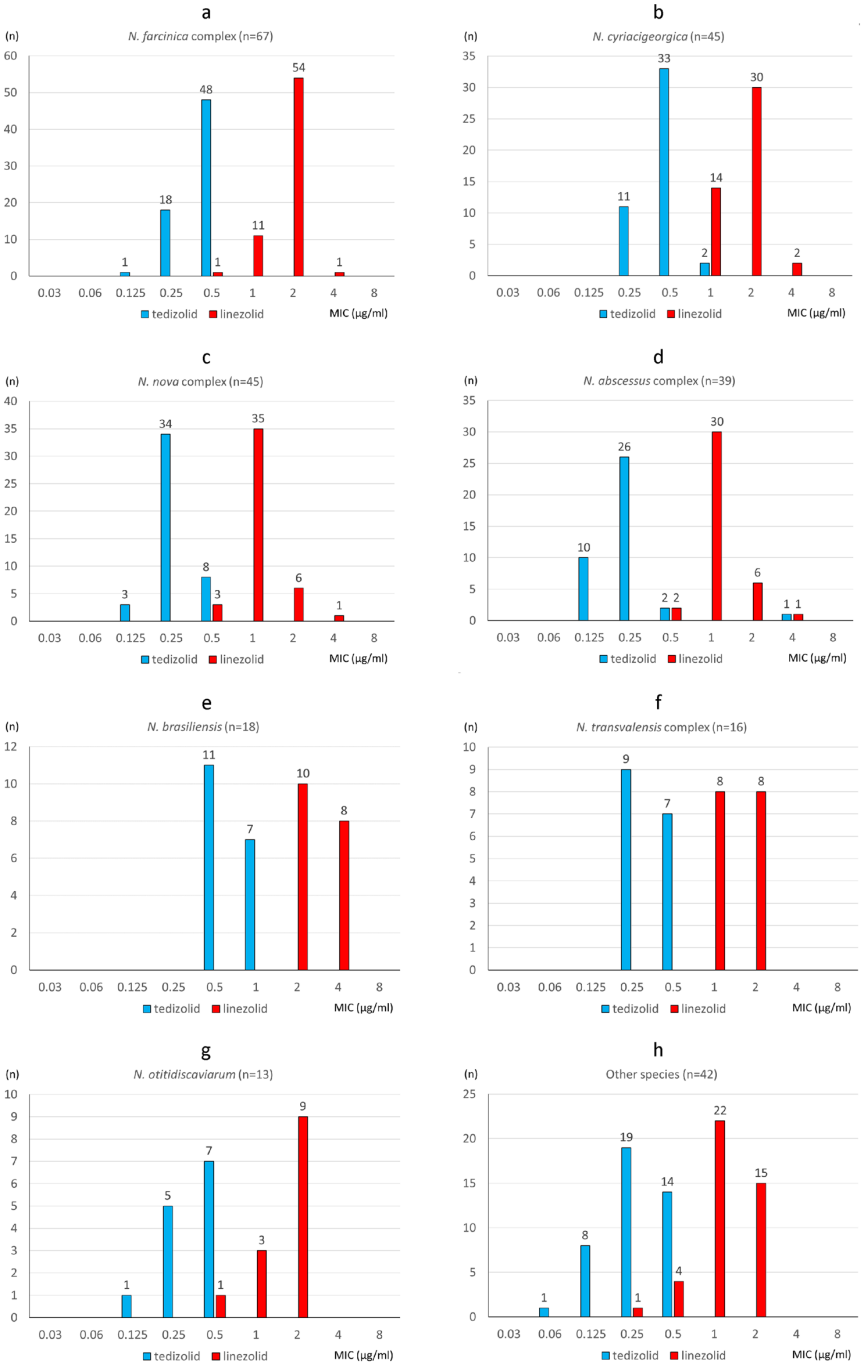
MIC distributions of tedizolid and linezolid against 286 *Nocardia* isolates. ^a^*N. farcinica* complex, ^b^*N. cyriacigeorgica*, ^c^*N. nova* complex, ^d^*N. abscessus* complex, ^e^*N. brasiliensis*, ^f^*N. transvalensis* complex, ^g^*N. otitidiscaviarum*, ^h^other species.

The MIC ranges, MIC_50_ values, MIC_90_ values and MIC distributions for tedizolid and linezolid against the 286 isolates of *Nocardia* species/complexes are shown in Table 2 and Fig. 1. For the 286 *Nocardia* isolates, the MIC_50_ and MIC_90_ values of tedizolid were 0.25 and 0.5 μg/ml, and those of linezolid were 2 and 2 μg/ml, respectively. Both the tedizolid and linezolid MIC_90_ values for *N. brasiliensis* were two-fold higher than those for the other *Nocardia* species. Both tedizolid and linezolid had low MIC ranges, 0.25–1 (mode = 0.5) μg/ml and 0.5–4 (mode = 2) μg/ml, respectively, even against the nine isolates that were resistant to trimethoprim/sulfamethoxazole isolates (three of *N. otitidiscaviarum*, two of *N. transvalensis* complex, two of *N. mexicana*, one of *N. cyriacigeorgica*, and one of *N. terpenica*). On the other hand, one clinical isolate of *N. abscessus* complex (*N. sputorum* IFM 12275) indicated higher MIC of tedizolid (4 μg/ml, measured three times on different days) than the other isolates (up to 1 μg/ml).

**Table 2:** MIC ranges, MIC_50_ and MIC_90_ values for tedizolid and linezolid against the 286 isolates of *Nocardia* species.

Species (no. of isolates tested) and antimicrobial	MIC (μg/ml)		
	Range	MIC_50_	MIC_90_
All (286 isolates)			
Tedizolid	0.06–4	0.25	0.5
Linezolid	0.25–4	2	2
*N. farcinica* complex^a^ (67 isolates)			
Tedizolid	0.12–0.5	0.5	0.5
Linezolid	0.5–2	2	2
*N. cyriacigeorgica* (46 isolates)			
Tedizolid	0.25–1	0.5	0.5
Linezolid	1–4	2	2
*N. nova* complex^b^ (45 isolates)			
Tedizolid	0.12–0.5	0.25	0.5
Linezolid	0.5–2	1	2
*N. abscessus* complex^c^ (39 isolates)			
Tedizolid	0.12–4	0.25	0.25
Linezolid	0.5–4	1	2
*N. brasiliensis* (18 isolates)			
Tedizolid	0.5–1	0.5	1
Linezolid	2–4	2	4
*N. transvalensis* complex^d^ (16 isolates)			
Tedizolid	0.25–0.5	0.25	0.5
Linezolid	1–2	1	2
*N. otitidiscaviarum* (13 isolates)			
Tedizolid	0.12–0.5	0.5	0.5
Linezolid	0.5–2	2	2
Other species (42 isolates)			
Tedizolid	0.06–0.5	0.25	0.5
Linezolid	0.5–2	1	2

^a^*N. farcinica* and *N. kroppenstedtii* were included in the *N. farcinica* complex, ^b^*N. africana*, *N. aobensis*, *N. elegans*, *N. kruczakiae*, *N. nova, N. vermiculata* and *N. veterana* were included in the *N. nova* complex, ^c^*N. abscessus*, *N. arthritidis*, *N. asiatica*, *N. sputorum*, and *N. beijingensis* were included in the *N. abscessus* complex, ^d^*N. blacklockiae*, *N. transvalensis*, and *N. wallacei* were included in the *N. transvalensis* complex.

**Figure 2:**
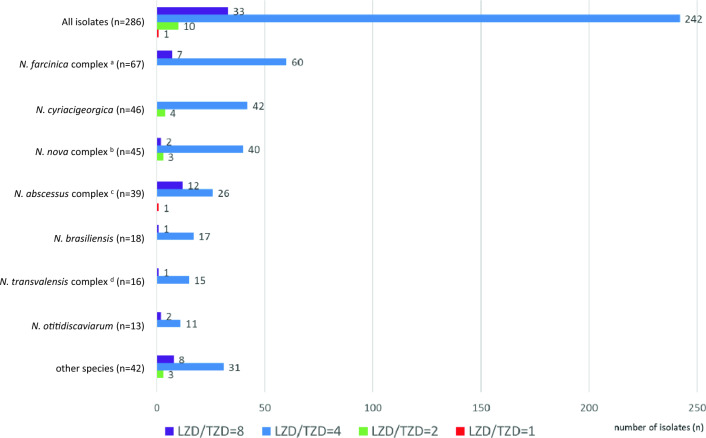
Distribution of the linezolid/tedizolid ratios (MICs of linezolid/MICs of tedizolid) against the 286 isolates of *Nocardia* species. ^a^*N. farcinica* and *N. kroppenstedtii* were included in the *N. farcinica* complex, ^b^*N. africana*, *N. aobensis*, *N. elegans*, *N. kruczakiae*, *N. nova, N. vermiculata* and *N. veterana* were included in the *N. nova* complex, ^c^*N. abscessus*, *N. arthritidis*, *N. asiatica*, *N. sputorum*, and *N. beijingensis* were included in the *N. abscessus* complex, ^d^*N. blacklockiae*, *N. transvalensis*, and *N. wallacei* were included in the *N. transvalensis* complex.

The distribution of the linezolid/tedizolid ratios (MICs of linezolid/MICs of tedizolid) against the 286 isolates of *Nocardia* species are shown in Fig. 2. Tedizolid was four- to eight-fold more active than linezolid in 96.1% (275/286) of *Nocardia* isolates. One clinical isolate of *N. abscessus* complex (*N. sputorum* IFM 12275) showed the same MIC value for linezolid (4 μg/ml) as that for tedizolid.

### Antimicrobial susceptibility patterns for the different *Nocardia* species

Antimicrobial susceptibility patterns for the different *Nocardia* species consisting of 236 clinical isolates and 50 NBRC strains are shown in Table 3. Only three drugs, amikacin, linezolid, and trimethoprim/sulfamethoxazole, showed ≥ 90% susceptibility among the all 286 isolates. The antimicrobial susceptibility patterns varied by species, and multidrug resistance (defined as four or fewer drugs that exhibit a susceptibility rate of 90% or higher) was common among *N. farcinica* complex, *N. nova* complex, *N. abscessus* complex, *N. transvalensis* complex, *N. otitidiscaviarum*, *N. thailandica*, *N. mexicana*, and *N. concava*.

**Table 3:** Antimicrobial susceptibility patterns for the different *Nocardia* species consisting of 236 clinical isolates and 50 NBRC strains.

Species (no. of isolates tested)	% of susceptible isolates of species or antibiotic susceptible pattern										
	Amikacin	Tobramycin	Imipenem	Ceftriaxone	Clarithromycin	Amoxicillin-clavulanic acid	Ciprofloxacin	Moxifloxacin	Minocycline	Linezolid	Trimethoprim-sulfamethoxazole ^e^
All (286)	**94.8**	51.4	74.1	44.8	23.1	48.3	16.4	34.6	21.7	**100**	**96.8**
*N. farcinica* complex^a^ (67)	**100**	1.5	86.6	4.5	1.5	**92.5**	32.8	61.2	6	**100**	**100**
*N. cyriacigeorgica* (46)	**100**	**100**	**100**	82.6	0	13	0	0	6.5	**100**	**97.8**
*N. nova* complex^b^ (45)	**100**	88.9	**97.8**	42.2	77.8	4.4	0	2.2	0	**100**	**100**
*N. abscessus* complex^c^ (39)	**100**	**100**	51.2	76.9	23.1	61.5	12.8	28.2	61.5	**100**	**100**
*N. brasiliensis* (18)	**100**	**94.4**	11.1	0	0	**94.4**	0	66.7	22.2	**100**	**100**
*N. transvalensis* complex^d^ (16)	25	0	75	81.3	18.8	81.3	37.5	50	6.3	**100**	87.5
*N. otitidiscaviarum* (13)	**100**	69.2	0	0	0	0	0	0	84.6	**100**	76.9
*N. takedensis* (3)	**100**	**100**	**100**	**100**	**100**	66.7	0	0	**100**	**100**	**100**
*N. thailandica* (3)	**100**	66.7	66.7	0	0	33.3	0	0	0	**100**	**100**
*N. asteroides* (2), *N. neocaledoniensis* (1)	**100**	**100**	**100**	**100**	0	0	0	0	0	**100**	**100**
*N. mexicana* (2)	50	0	50	**100**	0	0	0	0	0	**100**	0
*N. niigatensis* (2)	**100**	**100**	0	0	**100**	0	0	**100**	0	**100**	**100**
*N. pseudobrasiliensis* (2)	50	**100**	50	0	**100**	50	**100**	**100**	0	**100**	**100**
*N. vinacea* (2)	**100**	**100**	**100**	**100**	50	0	0	**100**	50	**100**	**100**
*N. yamanashiensis* (2)	**100**	0	0	0	**100**	0	0	**100**	0	**100**	**100**
*Nocardia* sp. (5)	**100**	**100**	80	80	20	20	80	**100**	20	**100**	**100**
*N. anaemiae* (1)	S^f^		S	S	S					S	S
*N. araoensis* (1)	S	S		S	S	S		S	S	S	S
*N. brevicatena* (1), *N. paucivorans* (1), *N. shimofusensis* (1)	S	S	S	S		S	S	S	S	S	S
*N. carena* (1)	S	S	S	S	S	S	S	S	S	S	S
*N. concava* (1)					S			S		S	S
*N. exalbida* (1)	S	S	S	S	S					S	S
*N. higoensis* (1)	S	S	S			S	S	S		S	S
*N. ignorata* (1)	S	S	S					S	S	S	S
*N. inohanensis* (1)	S	S	S	S	S	S		S	S	S	S
*N. ninae* (1)	S	S				S	S	S	S	S	S
*N. pneumoniae* (1)	S	S	S	S						S	S
*N. puris* (1)	S	S	S						S	S	S
*N. sienata* (1)	S	S	S	S			S	S		S	S
*N. terpenica* (1)	S	S			S			S		S	
*N. testacea* (1)	S	S	S				S	S		S	S
*N. uniformis* (1)	S	S	S	S		S			S	S	S

^a^*N. farcinica* and *N. kroppenstedtii* were included in the *N. farcinica* complex, ^b^*N. africana*, *N. aobensis*, *N. elegans*, *N. kruczakiae*, *N. nova, N. vermiculata* and *N. veterana* were included in the *N. nova* complex, ^c^*N. abscessus*, *N. arthritidis*, *N. asiatica*, *N. sputorum*, and *N. beijingensis* were included in the *N. abscessus* complex, ^d^*N. blacklockiae*, *N. transvalensis*, and *N. wallacei* were included in the *N. transvalensis* complex, ^e^Resistance to trimethoprim-sulfamethoxazole were determined by disk diffusion testing with a 250-μg sulfisoxazole disks. ^f^S, susceptible (only one isolate), Bold in the table indicates a susceptibility rate of 90% or higher.

### Minimum bactericidal concentration testing

The distribution of the MBC/MIC ratios of tedizolid and linezolid against the 23 clinical isolates of the seven *Nocardia* species showed that the MBC/MIC ratio was greater than or equal to eight for all 23 isolates.

### Analysis of resistance mechanisms in tedizolid resistant isolate

The ARGannot analysis on IFM 12275 resulted in hits for *ole*(C), *blab-4*, *aph*(3″), *tlr*(C), and *TlrC*, while CARD analysis resulted in hits for *vanY*, *vanW*, and rifampin monooxigenase, but acquired linezolid resistance genes known in enterococci, *cfr*, *cfr*(B), *cfr*(D), *optrA*, and *poxtA* were not detected by either method. On the other hand, the genes that were hits in the IFM 12275 strain other than *TlrC* were also detected in the IFM 12276^T^ strain. Of note, the *TlrC* gene has been reported as a gene associated with resistance for macrolide, lincosamide, and streptogramin group antibiotics^30^. In addition, in silico analysis by the Primer-blast confirmed that the acquired linezolid resistance gene described above was not detected in IFM 12275.

In the analysis of nucleotide mutations in the 23S rRNA gene (G2576T, G2505A, U2500A, G2447U, G2534U, G2603U), no mutations were observed in either of IFM 12275 and IFM 12276^T^ strains. Furthermore, the 50S ribosomal protein sequences of L3 (*rplC*), L4 (*rplD*), and L22 (*rplV*) were the same between the two strains. Comparing the gene sets in IFM12275 with those in IFM12276^T^, a total of 6285 orthologous genes were detected, and 500 and 387 unique genes were harboured in IFM12275 and IFM12276^T^, respectively.

## Discussion

Sulfonamides, mainly trimethoprim/sulfamethoxazole, have been the antimicrobials of choice to treat nocardiosis for a long time^4^. Trimethoprim/sulfamethoxazole still has a good activity against *Nocardia* species isolated in Japan^15^. However, adverse reactions to high-dose trimethoprim/sulfamethoxazole therapy, such as myelosuppression and hepatoxicity, are frequent^4^, and change in antibiotics is often necessary in such cases. Furthermore, the emergence of resistant bacteria due to prophylactic administration of low-dose trimethoprim/sulfamethoxazole has also been regarded as a problem. Averbuch et al. reported that the proportion of trimethoprim/sulfamethoxazole resistant *Nocardia* spp. was four of 25 (16%) among patients who received trimethoprim/sulfamethoxazole prophylaxis at the time of nocardiosis, compared with two of 36 (6%) among those who did not^2^.

Tedizolid has bacteriostatic activity against gram-positive cocci by binding to the 23S ribosomal RNA of the 50S subunit of the bacterial ribosome and inhibiting the early steps of bacterial protein synthesis^31^. Compared to linezolid, tedizolid shows stronger binding at the site of activity due to its unique D-ring substituent^31^; therefore, tedizolid is four- to 16-fold more potent in vitro than linezolid against many clinically relevant gram-positive cocci, including linezolid-resistant strains^26,32^. In the present study, we found that both tedizolid and linezolid had bacteriostatic activity (MBC/MIC ratio ≥ 8^21^) against *Nocardia* spp., and tedizolid was four- to eight-fold more active than linezolid in 96.1% (275/286) of *Nocardia* isolates. Brown-Elliott et al. reported that tedizolid had higher antibacterial activity in vitro than linezolid against 101 isolates of *Nocardia* species. They also cited the advantages of tedizolid over linezolid, including fewer serious adverse events, higher oral bioavailability, higher intracellular concentration, and a longer half-life, and speculated on its potential for the treatment of infections caused by *Nocardia* spp.^33^. There are some clinical case reports of the successful use of tedizolid for the treatment of disseminated nocardiosis^34–37^. In the four cases outlined in those case reports, all patients were initially treated with antimicrobial agents including linezolid, but tedizolid was used as an alternative due to myelosuppression caused by linezolid. In all cases, the patients were safely treated with tedizolid on prolonged treatment for two to six months without the development of myelotoxicity^34–37^. However, some studies have suggested the risk of thrombocytopenia with tedizolid^38^; further assessment is needed of thrombocytopenia development on prolonged treatment in the clinical setting.

On the other hand, in the present study, we found that one isolate, *N. sputorum* (IFM12275) showed higher MIC of tedizolid (4 μg/ml) than the other isolates (up to 1 μg/ml). The MIC of tedizolid for *N. sputorum* (IFM12275) was 16-fold higher than that for the same species, IFM12276^T^. Interestingly, this isolate had the same MIC value for linezolid (4 μg/ml). In the CLSI method, there is an interpretive breakpoint (BP) of linezolid against *Nocardia* species at 8 μg/ml, but no BP of tedizolid. Considering the BPs of linezolid and tedizolid are 4 μg/ml and 0.5 μg/ml for *S. aureus* and 2 μg/ml and 0.5 μg/ml for *Enterococcus faecalis*, respectively, the *Nocardia* isolate with MIC of 4 μg/ml in the present study is thought to be resistant to tedizolid. The mechanism of resistance to linezolid and tedizolid is being studied mainly in staphylococci and enterococci^12–14,22^. In these species, linezolid resistance is mediated through acquisition of resistance genes (*cfr*, etc.) or through ribosomal mutations in 23S rRNA gene or 50S ribosomal proteins L3, L4, and L22^12,22^. On the other hand, it has been reported that although tedizolid is active against linezolid-resistant stapylococci possessed *cfr* gene, it has cross-resistance to linezolid when mutations in chromosomal genes encoding 23S rRNA or ribosomal proteins (L3 and L4) are present^13,39^. Interestingly, it is also reported that the MICs of linezolid were four- to 16-fold higher than those of tedizolid, even in such cross-resistant strains to linezolid and tedizolid^12,14^, suggesting that the tedizolid-resistant strain in the present study with the same MIC values as linezolid, IFM 12275, is a very atypical strain.

Valdezate et al. reported two linezolid-resistant *N. farcinica* strains isolated from patients with cystic fibrosis^40^. These isolates indicated very high MIC of linezolid with ≥ 256 μg/ml. In their study, Valdezate et al. investigated the presence of genetic resistance determinants by PCR and a complete genome analysis of the strains, but they were unable to identify the resistance mechanism. Neither of the isolates harboured the *cfr* gene, nor did they show the mutations in 23S RNA (G2576T) allowing linezolid resistance, although the G2608A change was found in one allele of the one strain^41^. In the present study, we found that the tedizolid-resistant strain did not harbour the acquired linezolid resistance genes, nor did it show the mutations in 23S RNA and the 50S ribosomal protein. These results indicate that oxazolidinone resistance in *Nocardia* spp. is caused by a different resistance mechanism reported in enterococci and staphylococci. Since 500 genes were uniquely harboured in the tedizolid-resistant strain compared with the tedizolid-susceptible strain, another tedizolid-resistant strain would be helpful in identifying the candidate genes. Unfortunately, in the present study, we were unable to elucidate the mechanism of resistance to tedizolid in *Nocardia* species. Further research is necessary to elucidate the resistance mechanism and to set interpretive BP for tedizolid against *Nocardia* species.

A multicentric retrospective cohort study by Takamatsu et al. reported that the most frequently isolated *Nocardia* species in Japan were *N. farcinica* (79/317, 24.9%), *N. nova* complex (61/317, 19.2%), *N. abscessus* complex (59/317, 18.6%), and *N. cyriacigeorgica* (44/317, 13.9%)^41^. Identification of clinical isolates of *Nocardia* to the species/complex level is important, because *Nocardia* spp. differ in clinical spectrum and susceptibility patterns^42^. In the present study, differences in drug susceptibilities and multidrug resistance patterns were also observed depending on the *Nocardia* species. Recently, matrix-assisted laser desorption ionization-time of flight mass spectrometry (MALDI-TOF MS)-based identification has been identified as a rapid, easy, and reliable method^15,43,44^. Accurate identification by MALDI-TOF MS and antimicrobial susceptibility profiles together can help earlier implementation of empirical treatment and improvement of patient prognosis. On the other hand, we found one isolate which showed reduced susceptibility to tedizolid; in addition, linezolid-resistant clinical isolates have already been reported^40,45^, so performing antimicrobial susceptibility testing against all clinically significant isolates is recommended, especially in disseminated *Nocardia* infection.

In conclusion, tedizolid may be a useful treatment option for *Nocardia* infections, such as those in patients with trimethoprim/sulfamethoxazole allergies, and multidrug-resistant *Nocardia*.

### Supplementary Information

Supplementary Information.

## Data Availability

The datasets used and/or analysed during the current study available from the corresponding author on reasonable request.
